# Reply: Faecal tumour pyruvate kinase M2: not a good marker for detection of colorectal adenomas

**DOI:** 10.1038/sj.bjc.6604679

**Published:** 2008-09-30

**Authors:** U Haug, S Hundt, H Brenner

**Affiliations:** 1 Division of Clinical Epidemiology and Aging Research, German Cancer Research Center, Bergheimer Street 20, Heidelberg 69115, Germany

**Sir,**

We thank Shastri and Stein for their interest in our work and we appreciate the opportunity to clarify the issues raised by these authors:

1. As described in the MATERIALS AND METHODS section, the study includes participants of screening colonoscopy. Study participants who are covered by the statutory health care system are typically aged 55 years or above corresponding to the age at which screening colonoscopy is offered by this health care modality. The small proportion of study participants below the age of 55 (4.6%) years includes people covered by private health insurance, or people who undergo screening colonoscopy at their own expense.
2. The calculations of Shastri and Stein regarding the number of screening colonoscopies performed in the gastroenterological practices are strongly misleading for several reasons. First, the recruitment of practices started in January 2006, but most of them started to enrol patients at a later point of time. Some practices also participated in recruitment only for a short time period, but could not maintain their commitment because of work overload or organisational matters. Thus, the average recruitment period wa considerably less than 2 years. Second, not all people willing to participate in the study were eligible for the present analyses (see inclusion and exclusion criteria). Third, not all eligible people undergoing screening colonoscopy were willing to participate in the study.
3. Sufficient understanding of study information is an important prerequisite to allow for an informed consent, which is a fundamental principle of the Declaration of Helsinki. We did not have the resources to offer the study information in the variety of languages that would have been necessary to overcome this exclusion criterion. We do not think, however, that this point is of any relevance for our results, because only a very small proportion of patients had to be excluded owing to this criterion.

